# Assessment of nutritional and functional profile of whole, hulled and germinated hemp (*Cannabis sativa* L.) seeds

**DOI:** 10.3389/fnut.2026.1825902

**Published:** 2026-04-22

**Authors:** Daniel Mierlita

**Affiliations:** Laboratory of Nutrition, Department of Animal Science and Agritourism, University of Oradea, Oradea, Romania

**Keywords:** antioxidant activity, hemp seeds, nutrients, omega-3, polyphenols, protein digestibility

## Abstract

Hemp seeds (*Cannabis sativa* L.) have received considerable attention due to their nutrient and phytochemical content. However, while the nutritional and functional profile of whole hemp seeds has been adequately described in the literature, these aspects have not been investigated for hulled seeds, especially germinated hemp seeds. Therefore, the aim of this work was to explore the nutritional and functional profile of hulled seeds (DH) and germinated seeds (GH), compared to whole hemp seeds (WH), to elucidate their potential to be considered as viable alternatives for the food industry and animal feed. The proximal composition, concentration of antinutritional compounds, amino acid profile (AA), fatty acid profile (FA), tocopherol and phenolic content, and antioxidant activity were determined. Protein quality was assessed after *in vitro* digestibility was determined, and lipid quality indices were calculated. Compared to WH, hulled seeds had a higher content of crude protein (33.78% vs. 25.14%), crude fat (48.13% vs. 31.46%) and metabolizable energy (23.43 MJ kg−1 vs. 13.75 MJ kg−1), as well as the best *in vitro* protein digestibility (86.73% vs. 78.34%), which also ensured the best IVPDCAAS (*in vitro* protein digestibility corrected amino acid score) value. Seed germination resulted in a significant increase in protein, fiber and minerals, as well as in the content of antioxidant compounds, responsible for the higher antioxidant activity compared to WH and DH. In conclusion, hulling or germination improves the nutritional and functional profile of hemp seeds, confirming their potential for use in various emerging food matrices or in animal feed.

## Graphical abstract

**Figure:**
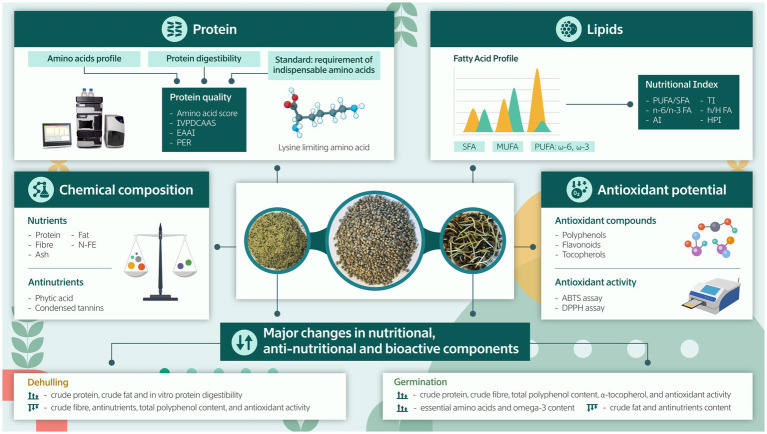

## 1 Introduction

Studies conducted to date indicate that, although whole hemp seeds (*Cannabis sativa* L.) are recognized for their high nutritional value and content of bioactive compounds, processes such as dehulling (removal of the hull) and germination may significantly alter their composition. Data on the nutritional and functional profile of hulled and germinated hemp seeds remain limited and fragmented, requiring more rigorous characterization to enable their full utilization in human and animal nutrition.

Hemp seeds contain approximately 21.3–32.0% protein, 25.4–35.9% oil, 27.8–38.8% fiber and 3.7–6.3% ash (1). In addition, they contain various bioactive components, including essential amino acids, omega-3 fatty acids (FA), tocopherols, carotenoids and phenolic compounds (2, 3).

Recent research has shown that hemp seed proteins contain all the amino acids (AA) that are essential for humans and animals (1). Detailed analyses have confirmed that the amino acid profile of hemp seeds is comparable to that of casein, soy protein or even egg white, with the exception of lysine, which is considered the first limiting amino acid (4, 5). This finding is especially important for animal nutrition and for growing children, for whom an adequate intake of lysine is essential. In addition, arginine, which plays a crucial role in blood pressure control, has been found to predominate in hemp seeds (6). Furthermore, numerous studies have reported that hemp seeds contain a significant amount of omega-3 FA and antioxidants, such as phenolic compounds, tocopherols, phytosterols and antioxidant peptides (3), which confer anti-inflammatory, antitumor and antineuroinflammatory effects (7). Therefore, hemp seeds can be considered a valuable and sustainable source of nutrients and bioactive compounds, with great potential to improve human health and support animal production and health.

However, the use of hemp seeds in food and feed is still limited by the low amount of lysine, low protein digestibility and presence of antinutritional components that reduce the digestibility and bioavailability of nutrients (6). Phytic acid, the most abundant antinutritional factor, forms insoluble complexes with divalent cations (Fe, Zn, Ca, Mg), such that the molar ratios of phytates to Fe (>20) and phytates to Zn (>15–35) far exceed the recommended thresholds, thereby compromising their absorption (2). Condensed tannins, primarily localized in the seed coat, interfere with digestive enzymes and precipitate proteins, reducing the bioaccessibility of amino acids (7). It has been shown that various processing methods, such as hulling, soaking, boiling, roasting and sprouting, can reduce the presence or activity of antinutritional compounds and improve the nutritional and functional profile of the plant seeds (8–11).

Hemp seeds used for direct consumption or in various food applications are often hulled, which involves removing the hard shell, because the hulls have a high content of antinutrients, including THC (tetrahydrocannabinol, a substance with psychoactive activity) (12). On the other hand, the hulls contain significant amounts of bioactive compounds (13), so the nutritional and functional profile of hulled seeds differ from those of whole (unhulled) seeds.

Germination has proven to be a simple and inexpensive process, capable of improving the nutritional and functional properties of the seeds of various plants. The germination of legume grains improves their nutritional value by increasing the levels of free amino acids and bioactive compounds and increasing the bioavailability of proteins and minerals, by reducing the contents of phytic acid and tannin (14). Recent studies (15) have reported significant improvements in protein content and quality, total phenolic compounds and antioxidant capacity following 3-day germination of chia seeds.

Limited information is available on the nutritional and functional profiles of germinated hemp seeds, including their macronutrient content, amino acid and fatty acid profiles, concentration of antinutritional compounds, and antioxidant content. Existing studies have mainly focused on whole hemp seeds, leaving the effects of hulling and germination on their nutritional and functional value to be elucidated. Knowledge of these effects may be essential not only from a nutritional point of view, but also regarding the potential beneficial effects of hulled or germinated hemp seeds for human and animal health beyond providing nutrients. Therefore, the aim of this study was to investigate the effect of economical physical treatments, such as hulling and germination, on the nutritional quality and antioxidant activity of hemp seeds. Our hypothesis was that hulling and germination could significantly impact their nutritional and phytochemical profiles. Furthermore, we anticipated that germinated hemp seeds could have greatly improved antioxidant activity. The results of the current study could provide important information for the better use of hemp seeds as a valuable source of vegetable protein with an improved amino acid profile, and a source of omega-3 fatty acids and antioxidants, for functional food applications and in animal feed.

## 2 Materials and methods

### 2.1 Plant material and germination

Hemp seeds (Jubileu variety) were provided by a local farmer in northwestern Romania. After harvest, the seeds were manually cleaned to remove plant debris and any other foreign matter. Dehulling was performed using an industrial dehuller (JHI05.2 PRO, JK Machinery a.s., Prague, Czech Republic), supplied by a local processor. The hulled hemp seeds were manually refined, with remaining hull fragments and unhulled seeds removed. The samples were packaged in dark-colored plastic bags and stored at −20 °C to prevent lipid oxidation and the photochemical degradation of antioxidants.

Because germination provides optimal conditions for microbial development (15), the hemp seeds, after being manually cleaned of impurities, were sanitized with sodium hypochlorite to reduce the microbial load (16). Next, the seeds were soaked in tap water (1,5, w/v) for 12 h at room temperature (20–22 °C). The seeds were allowed to germinate in plastic trays containing four layers of water-soaked filter paper. The trays were kept in the dark at 20–22 °C for 5 days (17) and watered daily by spraying. Microbial growth was monitored during seed germination by direct observation (the appearance of mold or unpleasant odors); germination was uniform, and the resulting sprouts exhibited a non-sticky texture and a fresh odor. Finally, the germinated seeds were frozen for 12 h to stop the germination process. After thawing at room temperature, the samples were dried in an electric oven (Isotemp Oven, Fisher Scientific) at 45 ± 2 °C for 18 h.

Whole seeds (unhulled or germinated) served as a control. All samples (WH, DH, GH) underwent identical processing steps: drying at 40 °C for 72 h, grinding using an electric grinder (Braun, Model 1,021, Germany), and storage in dark, airtight containers at 4 °C for subsequent analyses. Three replicates were performed for each analysis.

### 2.2 Chemical analyses

#### 2.2.1 Determination of proximate composition and energetical values

The AOAC (18) standard procedures were used to determine the dry matter (DM; AOAC 925.09), crude protein (CP; N x 6.25; AOAC 960.52), ether extract (EE; AOAC 2003.05), crude fiber (CF, AOAC 978.10) and crude ash (CA; AOAC 923.03) contents. Neutral detergent fiber (NDF) and acid detergent fiber (ADF) were analyzed according to the method described by Van Soest et al. (19), using an Ankom fiber analyzer (Ankom Technology, Macedon, NY, USA). The nitrogen-free extract (N-FE) was obtained by the difference (100 − (CP% + EE% + CF% + CA%)). The total carbohydrate content was determined by subtracting the percentages of protein, fat and ash from 100.

The gross energy (GE) and nitrogen-corrected metabolizable energy (AMEn) of the seed were calculated according to Wiseman et al. (20). AMEn was calculated according to Equation 1:


AMEn(kcal/kgDM)=3,951+54.4EE−88.7CF−40.8CA (1)

#### 2.2.2 Determination of antinutritional factors

Phytic acid was determined using the colorimetric method described by Wheeler and Ferrel (21), based on the precipitation of phytate as a ferric salt. Phytate was extracted using 3% trichloroacetic acid. The fraction was precipitated as ferric phytate, and the iron was released by boiling with 1.5 N NaOH solution for 30 min and subsequently dissolved with nitric acid. Quantitative determination of iron was carried out by measuring the absorbance at 480 nm. Calibration was performed using a ferric nitrate standard curve, and the phosphorus content of the phytate was calculated based on the specific molecular ratio of the precipitate (4:6). Finally, the phytic acid content was determined by multiplying the obtained phosphorus values by a factor of 3.55. The method was applied within the optimal operating range of 5 to 30 mg of phytic phosphorus.

Condensed tannins were determined using the vanillin assay described by Butler et al. (22). Briefly, tannins were extracted by mixing 0.5 g of sample with 5 mL of 4% HCl in methanol for 18 h, followed by centrifugation at 4,500 g for 10 min. For the colorimetric reaction, in a 96-well plate, the extract was mixed with 1% vanillin and 10% HCl in methanol. The mixture was incubated at room temperature for 10 min, and the absorbance was measured at 500 nm using a microplate reader. Calibration was performed using a (+)-catechin standard curve in the range of 0.25–1.0 mg/mL. The results were expressed as milligrams of catechin equivalents per 100 g DM. All analyses were performed in triplicate.

Although at least four types of antinutritional factors are present in hemp seeds, in the present study we assessed only the levels of phytic acid and condensed tannins, considering their dominant physiological impact, their relative concentrations, and the positive statistical correlations previously established among all antinutritional factors (23). Furthermore, since condensed tannins are predominantly located in the seed coat, their evaluation is essential to assess the efficiency of the dehulling process. The other antinutritional factors present in hemp seeds, such as trypsin inhibitors and saponins, are typically found at levels that do not pose a significant nutritional risk to humans or animals (23).

#### 2.2.3 Amino acid analysis

The amino acid (AA) composition was determined following acid hydrolysis (AOAC 982.30, 1995) using a Perkin Elmer Flexar-LC HPLC system (Waltham, MA, USA). Briefly, samples were hydrolyzed with 6 N HCl at 110 °C for 22 h under a nitrogen atmosphere. After hydrolysis, prior to injection, the released amino acids were filtered and derivatized with o-phthalaldehyde, except for proline, which was derivatized with fluorenylmethyl chloroformate. Sulfur-containing amino acids were determined after oxidation of the sample with performic acid followed by hydrolysis in 6 N HCl. Tryptophan was determined by alkaline hydrolysis using saturated barium hydroxide.

Chromatographic separation was performed using a reversed-phase Novapak C18 column (300 mm × 3.9 mm, 4 μm; Waters, Milford, MA, USA) with fluorescence detection (excitation at 270 nm, emission at 316 nm). Quantification was carried out by internal standard calibration using D, L-*α*-aminobutyric acid to normalize peak areas. Six-point linear calibration curves were established for each amino acid (R^2^ > 0.999). A detection limit of 0.20 μg/mL was established based on a signal-to-noise ratio (S/N) of 3, while the limit of quantification of 0.75 μg/mL was derived from an S/N ratio of 10 (2). The amino acid content of proteins in the analyzed samples was expressed as g/16 g N (equivalent to g/100 g protein) (24). All analyses were performed in triplicate.

#### 2.2.4 Protein quality assessment

Protein quality was assessed using different metrics such as the *in vitro* protein-digestibility corrected amino acid score (IVPDCAAS), essential amino acid index (EAAI) and protein efficiency ratio (PER).

To determine the *in vitro* protein digestibility (IVPD), the pH-decreasing method was used, as described by Nosworthy et al. (25). After treating the sample with deionized water (10 mL) and adjusting its pH (pH—8.0), a mixture of proteolytic enzymes (chymotrypsin, trypsin and protease) with an adjusted pH of 8.0 was added to the obtained protein solution. The pH was recorded for 10 min, at 30-s intervals. Based on the pH change after 10 min of enzyme action (∆pH10min: pH change compared to the initial pH), IVPD (%) was calculated according to the following Equation 2 (11).


IVPD(%)=65.66+18.10xΔpH10min (2)

To calculate the IVPDCAAS, the amino acid score (AAS) was multiplied by the IVPD (%). The AAS was determined by comparing the amino acid content of the investigated proteins with different reference standards. The amino acid ratio with the lowest value (below 1.00) was selected as the AAS.

To assess the protein quality of the investigated samples, we used five reference standards: the amino acid contents of whole egg proteins (26); the AA requirements for adult humans (24); the AA requirements for growing pigs weighing 20–50 kg (27); the AA requirements for 0-3-week-old broiler chickens; and the AA requirements for laying hens (28).

The essential amino acid index (EAAI) was calculated using the amino acid composition of the analyzed samples, compared to the concentration of a corresponding standard (in g/16 g N) according to the following Equation 3 (29).


$$\text{EAAI}=\sqrt[{n}]{(\frac{a_{1}}{a_{1s}})\times 100\times \ldots \ldots \ldots \times (\frac{a_{n}}{a_{\mathrm{ns}}})\times 100}$$ (3)

where an is the AA content in the protein tested and ans the AA content in the reference protein.

The protein efficiency ratio (PER_1_) was calculated using the following regression Equation 4 (29).


$$\mathrm{PE}R_{1}=0.06320\;[\begin{array}{l} \mathrm{Thr}+\mathrm{Val}+\mathrm{Met}+\mathrm{Ile}+\mathrm{Leu}+\\ \mathrm{Phe}+\mathrm{Lys}+\mathrm{His}+\arg+\mathrm{Tyr} \end{array}]-0.1539$$ (4)

The protein efficiency ratio (PER_1_) is traditionally expressed as the ratio of weight gain to the amount of protein consumed in rats. Because this method measures weight gain but not weight maintenance, it cannot be applied to mature humans. Accordingly, Amza et al. (29) proposed Equation 5 for calculating the PER_2_, expressed in terms of Leu and Tyr availability (g/16 gN):


$$\mathrm{PE}R_{2}=-0.468+0.45\mathrm{4Leu}-0.10\mathrm{5Tyr}$$ (5)

The predicted biological value (P-BV) and nutritional index (NI) of the analyzed samples were calculated using the following Equations 6, 7 (29).


BV=1.09(EAAI)–11.7 (6)

$$\text{Nutritional index}\;(\%)=\frac{\text{EAAI}\times \%\text{protein}}{100}$$ (7)

#### 2.2.5 Analysis of fatty acids and calculation of health lipid indices

Lipid extraction and fatty acid methyl ester (FAME) extraction were performed according to the method proposed by Christie (30). The fatty acid composition was determined by capillary gas chromatography using a Varian GC 3600 equipped with a flame ionization detector (FID), automatic injector and fused silica capillary column (SP 2560 Supelco, 100 m × 0.25 mm i.d., film thickness 0.20 μm; Varian, CA, USA). The carrier gas was helium, and the flow rate was 1 mL/min. The split ratio was 1:100. The initial oven temperature was set at 70 °C and maintained for 2 min, then increased to 190 °C at a rate of 8 °C/min and held at this level for 25 min. Subsequently, the oven temperature was increased by 3 °C/min to 230 °C and maintained for 7 min. The injector and detector temperatures were set at 270 °C (31). Fatty acid identification was carried out by comparing the retention times of the sample peaks with those of a standard mixture of fatty acid methyl esters (NuCheck Prep Inc., Elysian, MN, USA). All analyses were performed in triplicate.

The lipid quality indices of the investigated samples were calculated based on the proportions of certain FA, as follows: the ratio between saturated FA (SFA) and unsaturated FA (UFA); the ratio between n-6 and n-3 FA; the ratio between hypocholesterolemic and hypercholesterolemic FA (h/H); the atherogenic index (AI), thrombogenic index (TI), peroxidability index (PI), oxidative stability (OS), health-promoting index (HPI) and nutritional value indices (NVI) using established formulas (32, 33).

#### 2.2.6 HPLC analysis of tocopherols

A mixture of diethyl ether and petroleum ether (1:1) was used to extract tocopherols, and a methanolic solution of KOH (10%) was used to saponify the ether extract. After saponification, extraction in hexane followed, followed by washing with water in a separatory funnel and evaporation to dryness (3).

Separation and quantification of tocopherols were performed using a Perkin-Elmer LC-295 HPLC system equipped with an Alltech C18 column (15 cm × 4.6 mm i.d., 3 μm particle size). The mobile phase consisted of a mixture of acetonitrile:methanol (85:15) and isopropanol (90:10). The mobile phase flow rate was set at 1.0 mL/min, the column temperature was maintained at 25 °C, and the injection volume was 20 μL. Detection was carried out fluorimetrically, with an excitation wavelength of 295 nm and an emission wavelength of 330 nm. Compound identification was performed based on retention times, with typical values of approximately 5 min for *α*-tocopherol, 7 min for *γ*-tocopherol, and 9 min for *δ*-tocopherol. Calibration was carried out using standard solutions of tocopherols in the concentration range of 0.5–20 μg/mL, prepared in the mobile phase. Calibration curves were constructed by plotting peak area versus concentration (R^2^ > 0.999). The limit of detection and limit of quantification were approximately 0.05 μg/mL and 0.15 μg/mL, respectively. Using Millennium 3.2 software, each isomer was identified by comparing the retention times of sample peaks with those obtained from pure standards. Based on the chromatograms of the four standard solutions, the ratio between the peak area of the analyzed tocopherol and the peak area of the internal standard (rac-Tocol) was calculated. By applying the calibration curve equation to the normalized peak area of the unknown samples, the concentration of each isomer in the sample was determined. All analyses were performed in triplicate. Total Phenolic Content and Antioxidant Activity Analyses.

The ground sample (3 g) was treated with a mixture of methanol/water (80:20, v/v) in a ratio of 1:10 (v/w). Then, the samples were centrifuged for 15 min at 4 °C (Sigma 2-16KL Refrigerated Centrifuges, Berlin, Germany) after previously being processed in an orbital shaker (GFL 3005, GEMINI, Apeldoorn, The Netherlands) for 30 min. The supernatants obtained were passed through a nylon filter with a pore diameter of 0.45 μm.

TPC was determined using the Folin–Ciocalteu method. Briefly, a 20 μL aliquot of methanolic extract was mixed with 100 μL of Folin–Ciocalteu reagent (diluted 1:10 v/v) and 80 μL of sodium carbonate solution (7.5% w/v). The samples were incubated at room temperature, in the dark, for 60 min, followed by an additional incubation step at 40–45 °C for 30 min to complete the color development reaction. Absorbance was measured at 750 nm. Quantification was performed by interpolation on a calibration curve using gallic acid (R^2^ > 0.998), and the results were expressed as milligrams of gallic acid equivalents per 100 g of dry matter (mg GAE/100 g DM).

TFC was quantified using the aluminum chloride colorimetric method. A volume of 24 μL of extract was sequentially reacted with 28 μL of sodium nitrite (50 g/L) for 5 min and 28 μL of aluminum chloride (100 g/L) for 6 min. The reaction was stopped by adding 120 μL of sodium hydroxide (1 M), followed by brief mixing for 30 s. Absorbance was recorded at 510 nm. Calibration was performed using (+)-catechin as an external standard in the concentration range of 0.25–1.0 mg/mL (R^2^ > 0.999). The values were reported as milligrams of catechin equivalents per 100 g of dry matter (mg CE/100 g DM). All analyses were performed in triplicate.

The antioxidant activity was determined spectrophotometrically, using radical-scavenging methods (2,2-diphenyl-1-picrylhydrazyl (DPPH) and 2,2-azino-bis (3-ethylbenzothiazoline-6-sulphonic acid) (ABTS)), according to the procedure described previously (3).

The radical scavenging activity against 2,2-diphenyl-1-picrylhydrazyl (DPPH) was evaluated according to the method described by Brito et al. (34), with slight modifications. Briefly, 10 μL of extract were mixed with 190 μL of freshly prepared DPPH solution (0.2 mmol/L in methanol). The samples were incubated in the dark at room temperature for 30 min, after which the absorbance was measured at 517 nm. Seventy percent ethanol was used as the blank. Quantification was performed by interpolation on a Trolox calibration curve (range 150–750 μM; R^2^ > 0.998), and the results were expressed as milligrams of Trolox equivalents (mg TE/100 g DM).

The ABTS assay was performed by first preparing an ABTS working solution through the reaction of an ABTS solution (7 mM) with potassium persulfate (2.45 mM), the mixture being kept in the dark for 12–16 h prior to use. The solution was then diluted with ethanol to an absorbance of 0.70 ± 0.02 at 734 nm. A volume of 12 μL of extract was mixed with 188 μL of ABTS working solution, and the absorbance was read at 734 nm after 10 min at 25 °C. The results were calculated based on a Trolox standard curve (range 300–1,500 μM; R^2^ > 0.998) and expressed as mg TE/100 g DM of seed. All analyses were performed in triplicate.

### 2.3 Statistical analysis

All analyses were performed in triplicate (*n* = 3) for each sample (WH, DH, and GH) and for each type of determination (proximate composition, antinutritional compound content, amino acids, fatty acids and antioxidants, *in vitro* protein digestibility, and antioxidant activity). The obtained results were analyzed using one-way ANOVA, employing the SPSS statistical package version 18.0 (SPSS Inc., Chicago, IL, USA). Prior to analysis, the data were subjected to the Shapiro–Wilk test to verify normality of distribution and the Levene test to confirm homogeneity of variances. Mean separation was performed using Duncan’s multiple range test at a significance level of *p* < 0.05. The results are reported as mean ± standard deviation (SD). Additionally, Pearson correlations were calculated between antioxidant compounds and the antioxidant activity of hemp seeds.

## 3 Results and discussion

### 3.1 Chemical compositions and energy value

The seed-hulling process consists of removing the hulls, which should theoretically lead to (a) an increase in protein and fat content and (b) a decrease in other components such as crude fiber, NDF and ADF (8). Indeed, in the present study, DH had 34.3 and 52.9% increases (*p* < 0.01) in protein and fat content, respectively, compared to WH (Table 1). This change in chemical composition also led to an increase in the energy value of the DH samples compared to WH (23.43 MJ/kg vs. 13.75 MJ/kg). DH had significantly higher protein and fat contents, as both protein and fat are predominantly located in the kernels of hemp seeds and only a small amount is present in the shell (1, 35). The values found in this study for protein content were higher than those previously reported by Alonso-Esteban et al. (2), both for WH (25.1 g/100 g vs. 20.4 g/100 g) and for dehulled seeds (33.78 g/100 g vs. 26.0 g/100 g). The discrepancies compared to the study by Alonso-Esteban et al. (2) arise from the conversion factor used for the nitrogen-to-protein ratio (6.25 vs. 5.3), with our values remaining comparable to other studies that use the standard factor of 6.25 (36, 37).

**Table 1:** Proximate composition and energy value of whole (WH), dehulled (DH), and germinated (GH) hemp seeds (g/100 g of dry matter).

Parameters	WH	DH	GH
Dry matter (DM)	92.88 ± 2.34^ab^	94.96 ± 1.64^a^	88.90 ± 1.39^b^
Crude protein (CP) (N x 6.25)	25.14 ± 1.93^c^	33.78 ± 4.15^a^	28.24 ± 1.28^b^
Ether extract (EE)	31.46 ± 2.87^b^	48.13 ± 5.38^a^	26.44 ± 0.79^c^
Crude fibre (CF)	24.62 ± 1.26^b^	7.92 ± 0.71^c^	30.72 ± 1.81^a^
NDF (neutral detergent fiber)	32.86 ± 3.05^b^	7.02 ± 2.08^c^	37.18 ± 1.58^a^
ADF (acid detergent fiber)	23.31 ± 2.51^b^	5.25 ± 1.51^c^	27.75 ± 2.07^a^
Crude ash (CA)	4.69 ± 0.82^b^	6.54 ± 0.83^a^	6.28 ± 0.12^a^
N-FE (nitrogen-free extract)	14.09 ± 0.51^a^	3.63 ± 0.18^c^	8.32 ± 0.31^b^
Total carbohidrați	38.71	12.84	40.04
Gross energy (GE, MJ/kg)	19.29 ± 0.63^b^	25.26 ± 0.92^a^	18.81 ± 0.86^b^
AMEn (MJ/kg)	13.75 ± 0.42^b^	23.43 ± 0.82^a^	10.24 ± 0.40^c^

AMEn, nitrogen-corrected apparent metabolizable energy; Data are presented as mean ± standard deviation (SD) (*n* = 3). Values within a row not marked with the same superscript letter indicate significant differences according to Duncan’s multiple range test (*p* < 0.05).

Some studies have reported higher fat contents in DH [52.6% in (2); 51.17% in (36)] than those found in the present study (48.13%), while other studies have reported lower values [46.7%; (38)]. These differences may be associated with variations in hemp varieties, cultivation conditions or environmental conditions, with higher temperatures and lower precipitation leading to a decrease in the fat content of hemp seeds (7). Compared to WH, germinated seeds showed a higher protein content (*p* < 0.05), higher crude fiber (*p* < 0.01), lower fat (*p* < 0.05) and a lower energy value (*p* < 0.05) (Table 1).

The increase in protein and decrease in lipids during germination have also been reported in other plant seeds, such as lentils, peas, chickpeas, alfalfa, radish (39) and chia (40). The increase in protein content has been attributed to the synthesis of enzymatic proteins or the degradation of other constituents and reductions in their concentrations (41), while the decrease in lipid content during germination is due to the increase in lipolytic activity and the release of fatty acids, which are used as an energy source for the synthesis of DNA, RNA, enzymes and structural proteins and in other germination-specific metabolic activities (40).

The carbohydrates in hemp seeds are found predominantly in the seed coat (35, 42). Consequently, DH had significantly lower crude fiber, NDF, ADF and N-FE content (*p* < 0.01) compared to WH. Although the available data on carbohydrate concentration in DH are limited, the values found in this study for the average carbohydrate content are similar to those reported by Hwang et al. (1).

Germinated (GH) samples showed the highest crude fiber, NDF and ADF content compared to WH and DH samples. This is in agreement with other studies (41) which found that the fiber content increased in germinated barley seeds and lentil seeds. The N-FE content decreased from 14.09% in raw samples (WH) to 8.32% in germinated samples (GH). This decrease could be due to the increase in alpha-amylase activity, which breaks down complex carbohydrates into simple sugars, which are used in the germination process as energy sources (14). The NDF values obtained in the current study indicate that 85 and 93% of the total carbohydrate fraction in WH and GH, respectively, is represented by fiber, suggesting a low glycemic index of whole hemp seeds and hemp germs, due to the low percentage of non-fibrous carbohydrates present (38).

In our study, we found a significant (*p* < 0.05) increase in mineral content, as measured by crude ash content, in hemp seeds processed by dehulling and germination. Increased mineral content has also been observed in sprouts from other plant species, such as broccoli, zucchini, canola and amaranth (43). Furthermore, the present study confirms previous data regarding the amount of crude ash in WH and DH (2, 5).

### 3.2 Antinutritional compounds

**Figure 1:**
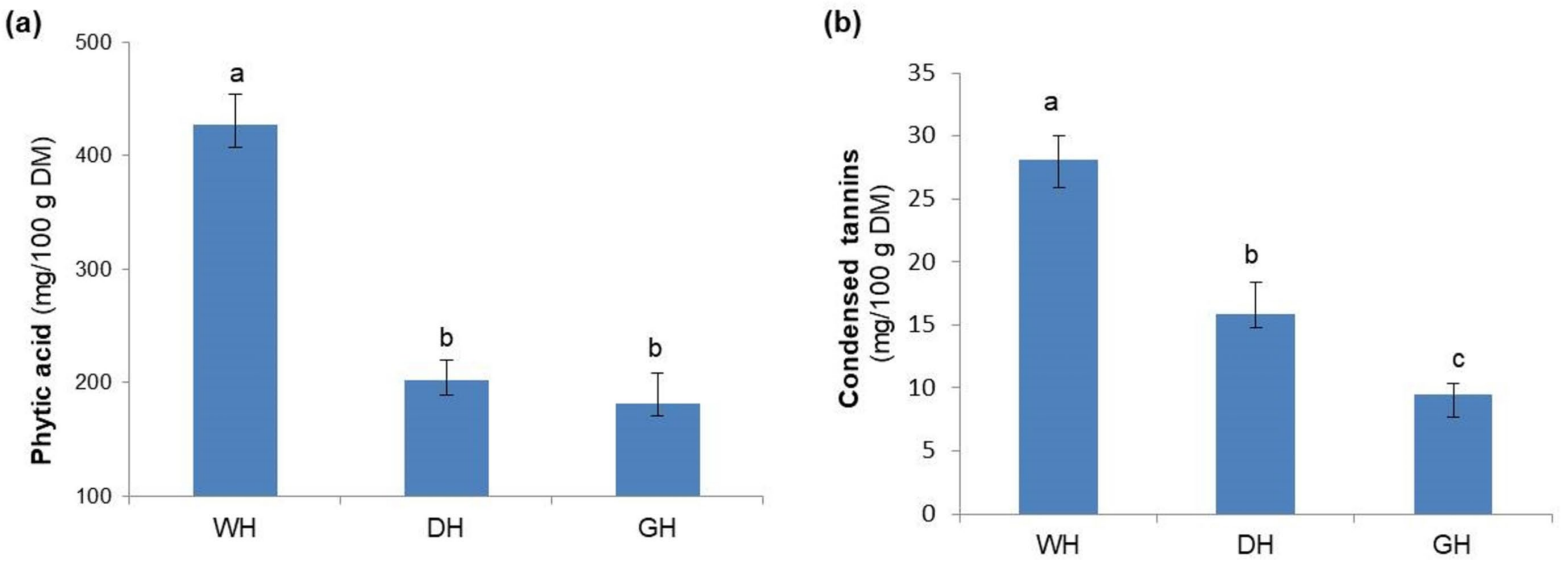
Phytic acid **(a)** and condensed tannin content **(b)** of the whole (WH), dehulled (DH), and germinated (GH) hemp seeds. Data are plotted as mean ± SD (*n* = 3). Statistical analysis was conducted using one-way ANOVA followed by Duncan’s multiple range test. Different letters above the bars indicate significant differences (*p* < 0.05).

Shelling and germination significantly decreased (*p* < 0.001) the concentration of antinutritional compounds, such as phytic acid and condensed tannins, in hemp seeds (Figure 1). WH samples had the highest phytic acid content (427.52 mg/100 g DM), followed by DH (201.68 mg/100 g DM) and GH (181.84 mg/100 g DM), respectively. The phytic acid content of the analyzed samples is well below the level reported for many legumes, such as lentils (1,075 mg/100), beans (1,224 mg/100 g) and chickpeas (1,114 mg/100 g) (44), but higher than that reported for soybeans or rapeseed (45).

Phytic acid mainly binds divalent minerals through the chelating action of its phosphate groups, but also binds proteins, reducing their bioavailability (15). A standard serving of hemp seeds (recommended at 30 g) provides 128.2 mg of phytic acid, out of the total 400–800 mg/day considered optimal for minimal impact on mineral absorption (46). Through dehulling or germination of hemp seeds, the phytic acid content is reduced by approximately half (60.5 mg and 54.5 mg phytic acid per serving, respectively), which could contribute to increased availability and utilization of minerals from diet. These observations are consistent with previous studies demonstrating that processing legume seeds using various techniques—such as cooking, fermentation, germination, and hull removal—leads to a reduction in phytic acid concentration (41, 47).

Tannins are considered antinutritional compounds because they form stable complexes with proteins and minerals, reducing their availability. At the same time, tannins can also have beneficial effects because their phenolic rings have antioxidant properties (7).

Among the analyzed samples, the highest tannin content was found in WH (28.14 mg/100 g DM), while dehulling and seed germination reduced the concentration of these compounds by 43.6 and 66.3%, respectively. The amount of condensed tannins in the investigated samples is higher than in soybean (45), which it can substitute in the human diet, but relatively low compared to that in animal feed (8), which suggests negligible nutritional interference for animals.

Because tannins and phytic acid are mainly located in the seed coat (12), it was expected that hulled seeds would contain lower amounts of these compounds. The reduction in phytate content during germination may be attributed to the increase in phytase activity, while the reduction in tannins may be due to the increase in polyphenol oxidase enzyme activity, resulting in the degradation and loss of tannins (48). In conclusion, processing hemp seeds by hulling or germination may improve the availability and utilization of nutrients, contributing to increasing their nutritional value and potential for use in food systems, to promote a healthy and sustainable diet.

### 3.3 Amino acid profile and protein nutritional quality

The data presented in Table 2 indicate that the total essential amino acids (EAA) in the WH, DH and GH proteins constituted 37.55, 38.41 and 39.32% of the total amino acid content, respectively. The protein in the analyzed samples was rich in essential amino acids, such as Met + Cys, Ile, Trp, Val, Leu, and Phe + Tyr compared to the FAO/WHO (24) reference. The essential amino acid scores (AAS), which compare the concentrations of less abundant amino acids with a standard, showed that Lys was the first limiting amino acid for all the analyzed hemp samples, followed by Thr, and the third limiting amino acid was Leu or Val, depending on the nutritional standard used (humans and animals: pig, broiler chicken and laying hen) (Table 3). However, Russo and Reggiani (23) reported that the first limiting AAs in hemp seeds are sulfur-containing amino acids (Met + Cys), followed by Lys, and in another study (38), Lys was found to be the first limiting amino acid, followed by Trp and Leu. Surprisingly, in our study, the content of Trp and His in the investigated proteins was well above the nutritional standards used, being similar to the level of these AAs in egg protein, which is a protein with high biological value. These differences can be attributed to genotype, environmental factors and different agronomic practices. However, the EAA content of the proteins in the investigated samples was much lower than that of egg white (37.55–39.22 g/16 g N vs. 51.2 g/16 g N), used as an amino acid standard in protein evaluation, which confirms that hemp proteins are lower quality than animal protein. However, hemp seeds have a higher essential amino acid (EAA) content than potato, wheat, corn, rice, rapeseed, and kidney bean but lower than soybean, which has a higher lysine content but lower levels of sulfur-containing amino acids (methionine and cysteine) (1, 4, 48). Although processing (dehulling and germination) improves the EAA profile of hemp seeds, it does not reach the EAA density of egg protein. Nevertheless, they surpass most plant-based and germinated seed sources (horse gram, flax, rapeseed, chia, broccoli) (14, 15, 40, 43), with the exception of quinoa seeds, which after 6 days of germination come closest to the egg profile, reaching an EAA content of 40.80–41.42 g/16 g N (49).

**Table 2:** Amino acid (AA) profile/content in the whole (WH), dehulled (DH), and germinated (GH) hemp seeds.

AAs	Content in protein (g/16 g N)			Content in seeds (g/100 g DM)		
	WH	DH	GH	WH	DH	GH
Essential amino acids (EAA)						
Lys	3.64 ± 0.21^b^	3.86 ± 0.12^ab^	3.94 ± 0.43^a^	0.92 ± 0.11^b^	1.30 ± 0.15^a^	1.11 ± 0.17^ab^
Met + Cys	3.42 ± 0.23^b^	3.69 ± 0.51^a^	3.18 ± 0.33^c^	0.86 ± 0.09^b^	1.25 ± 0.17^a^	0.93 ± 0.12^b^
Cys	1.40 ± 0.06	1.55 ± 0.04	1.32 ± 0.18	0.35 ± 0.06^b^	0.52 ± 0.07^a^	0.37 ± 0.06^b^
Thr	3.54 ± 0.14	3.51 ± 0.20	3.14 ± 0.31	0.89 ± 0.12^b^	1.19 ± 0.16^a^	0.89 ± 0.10^b^
Ile	3.87 ± 0.09	3.91 ± 0.13	3.66 ± 0.07	0.97 ± 0.13	1.32 ± 0.19	1.03 ± 0.08
Trp	1.71 ± 0.12	1.85 ± 0.36	1.64 ± 0.28	0.43 ± 0.06	0.63 ± 0.08	0.46 ± 0.04
Val	4.77 ± 0.52^b^	4.68 ± 0.38^b^	6.14 ± 0.61^a^	1.20 ± 0.15^b^	1.58 ± 0.18^ab^	1.73 ± 0.14^a^
Leu	6.43 ± 0.47	6.45 ± 0.64	6.82 ± 0.38	1.62 ± 0.17^b^	2.18 ± 0.24^a^	1.93 ± 0.18^a^
His	2.68 ± 0.08	2.60 ± 0.05	2.43 ± 0.07	0.67 ± 0.07	0.88 ± 0.05	0.69 ± 0.08
Phe + Tyr	7.49 ± 0.14^b^	7.86 ± 0.11^ab^	8.27 ± 0.14^a^	1.88 ± 0.12^b^	2.66 ± 0.22^a^	2.34 ± 0.09^a^
Tyr	3.02 ± 0.18	3.07 ± 0.07	3.52 ± 0.10	0.76 ± 0.09^b^	1.04 ± 0.14^a^	0.81 ± 0.06^b^
∑EAA	37.55 ± 0.74^b^	38.41 ± 1.15^ab^	39.22 ± 0.55^a^	9.44 ± 0.16^b^	12.98 ± 0.12^a^	11.10 ± 0.17^ab^
Non-essential amino acids (NEAA)						
Arg	10.31 ± 0.29	10.84 ± 0.75	10.57 ± 0.58	2.59 ± 0.04^b^	3.66 ± 0.12^a^	2.99 ± 0.23^b^
Asp	9.22 ± 0.37	9.41 ± 0.61	9.86 ± 0.45	2.32 ± 0.18^b^	3.18 ± 0.09^a^	2.78 ± 0.14^ab^
Ser	4.76 ± 0.21	4.43 ± 0.30	4.91 ± 0.64	1.20 ± 0.13^b^	1.50 ± 0.17^a^	1.39 ± 0.011^a^
Glu	14.17 ± 0.54	14.82 ± 0.39	14.67 ± 0.83	3.56 ± 0.29^b^	5.00 ± 0.16^a^	4.14 ± 0.23^ab^
Pro	3.34 ± 0.32	3.35 ± 0.29	3.20 ± 0.17	0.84 ± 0.12^b^	1.13 ± 0.13^a^	0.90 ± 0.12^b^
Gly	4.36 ± 0.41	4.31 ± 0.38	4.15 ± 0.26	1.10 ± 0.06^b^	1.46 ± 0.15^a^	1.17 ± 0.21^b^
Ala	4.32 ± 0.14	4.55 ± 0.21	4.81 ± 0.08	1.09 ± 0.14^b^	1.54 ± 0.07^a^	1.36 ± 0.17^ab^
∑NEAA	50.48 ± 1.26^b^	51.71 ± 1.95^a^	52.17 ± 0.78^a^	12.69 ± 0.45^c^	17.47 ± 0.52^a^	14.73 ± 0.47^b^
Total AA	88.03 ± 2.41^b^	90.12 ± 3.54^a^	91.39 ± 1.73^a^	22.13 ± 0.63^c^	30.44 ± 0.40^a^	25.84 ± 0.94^b^

Data are presented as mean ± standard deviation (SD) (*n* = 3). Values within a row not marked with the same superscript letter indicate significant differences according to Duncan’s multiple range test (*p* < 0.05).

**Table 3:** Evaluation of protein quality in whole (WH), dehulled (DH), and germinated (GH) hemp seeds, using different reference standards.

Parameters	WH	DH	GH
Standard: whole egg protein (26) (Lys—7, Met+Cys—5.7, Thr—4.7, Ile—5.4, Trp—1.7, Val—6.6, Leu—8.6, His—2.2, Phe + Tyr—9.3).			
AAS (%) (Lys)	0.520	0.551	0.563
EAAI (%)	76.38	78.34	77.26
P-BV (%)	71.55	73.69	72.51
Nutritional index (%)	19.20	26.46	21.81
Standard: nutrient requirement for mature human (24) (Lys—5.5, Met+Cys—3.5, Thr—4, Ile—4, Trp—1, Val—5, Leu—7, Phe + Tyr—6).			
AAS (%) (Lys)	0.661	0.702	0.716
EAAI (%)	100.40	103.51	102.80
P-BV (%)	97.74	100.76	100.35
Nutritional index (%)	25.24	34.96	29.03
Standard: nutrient requirement for growing pigs 20–50 kg (27) (Lys—7, Met+Cys—3.6, Thr—4.5, Ile—4, Trp—1.2, Val—5.2, Leu—8, His—2.5, Phe + Tyr—8).			
AAS (%) (Lys)	0.520	0.551	0.563
EAAI (%)	90.15	92.25	90.99
P-BV (%)	86.56	88.85	87.48
Nutritional index (%)	22.66	31.16	25.69
Standard: nutrient requirement of 0–3 weeks chicken broilers (28) (Lys—4.8, Met+Cys—3.9, Thr—3.5, Ile—3.5, Trp—0.9, Val—3.9, Leu—5.2, His—1.5, Phe + Tyr—3.9).			
AAS (%) (Lys)	0.758	0.804	0.821
EAAI (%)	124.73	127.64	125.89
P-BV (%)	124.26	127.43	125.52
Nutritional index (%)	31.35	43.12	35.55
Standard: nutrient requirement of layer hens (28) (Lys—4.6, Met+Cys—3.8, Thr—3.1, Ile—4.3, Trp—1.0, Val—4.6, Leu—5.4, His—1.1, Phe + Tyr—5.5).			
AAS (%) (Lys)	0.791	0.839	0.856
EAAI (%)	119.67	122.74	121.34
P-BV (%)	118.74	122.08	120.56
Nutritional index (%) (NI)	30.08	41.46	34.27
Lys/Arg	0.354	0.356	0.373
PER_1_	2.736	2.597	2.674
PER_2_	2.134	2.138	2.259

AAS, amino acid score; EAAI, essential amino acid index; PER, protein efficiency ratio. AAS, EAAI, BV, NI, Lys/Arg and PER are calculated values, no standard deviation is available.

The total amount of non-essential amino acids (NEAA) represented 50.48–52.17% of the total amino acid content. Glutamic acid, arginine and aspartic acid were found to be the main non-essential amino acids in the protein sources investigated in this study. This suggests that the use of hemp proteins in the diet may benefit cardiovascular health, because arginine serves as a precursor for the formation of nitric oxide, which acts as an important vasodilator for maintaining blood pressure and regulating blood flow (1). Consequently, the high arginine content makes the analyzed hemp products a valuable ingredient in the development of foods that promote cardiovascular health (4).

Although dehulling increased the total protein content, the relative concentrations of amino acids (g/16 g N) showed no significant variations (*p* > 0.05), except for sulfur-containing amino acids (*p* < 0.05). However, the absolute amino acid content (g/100 g DM) was significantly higher in the dehulled (DH) samples compared to the whole (WH) ones (*p* < 0.05) (Table 2). In contrast, the germination process caused significant increases (*p* < 0.05) in the contents of EAA and mainly Val, Lys and Phe + Tyr, but caused a significant decrease in the content of sulfur AA (Met + Cys). No information on the amino acid profile of germinated hemp seeds was found; however, Fouad and Rehab (41) reported results similar to those of the present study, namely a significant increase in essential amino acids, except for sulfur AA, in germinated lentil seeds. Interestingly, the germination of hemp seeds resulted in an increase in EAA of only 3.81%, compared to in the study by Barakat et al. (49), who found an increase ranging from 7.43 to 14.36% in quinoa seeds germinated for 6 days. This improvement in the AA profile can be explained by the fact that, during germination, proteases that cleave peptide bonds between amino acids are activated (49), and in addition, new protein synthesis processes occur, necessary for germ growth (14).

The amino acid score (AAS) of a protein reflects the extent to which an organism’s individual amino acid requirement is met. The lowest score is considered the AAS for the entire protein source, regardless of the relative contributions of the other amino acids (38). In our study, the highest AAS values were calculated for GH, followed by DH, and the lowest values were found for WH. The AAS values were 0.52 and 0.66 for WH protein, when the reference standard used was egg protein or the AA requirements for adult humans (24). These values were higher than those previously determined by Nosworthy et al. (10) (0.46 and 0.50), but lower than those established by Russo and Reggiani (23) for dioecious and monoecious hemp varieties (0.69 and 0.79). At the same time, the AAS values found in this study for DH were 0.55 and 0.70, similar to those in previous studies [0.57 to 0.72; (50)]. The differences can be attributed to several factors, such as varietal (genetic) differences, and agronomic and environmental conditions (precipitation, temperature) (50).

The AAS values of the proteins analyzed were higher for poultry (broiler chickens and laying hens) than for humans and growing pigs (Table 3), and this suggests that hemp proteins are more suitable for poultry nutrition. It should be noted that, to date, no studies have been conducted to evaluate the quality of hemp proteins using the AA requirements of animals (pigs, broilers and laying hens) as reference standards, so the results obtained in the present study could not be compared.

In general, the quality of a protein is influenced by the first limiting amino acid, but from a practical point of view, dietary protein sources are mixed in a diet precisely to correct for amino acids deficient in a particular protein source. It follows that, in the case of the proteins tested in the present study, their association with protein sources with a higher lysine content, such as soy proteins for animals and animal proteins for humans, will improve the overall amino acid score of the diet.

The content of essential amino acids (EAA) is reflected in the EAAI values which, in relation to the reference standard, were higher in DH samples than in WH (DH ˃ GH ˃ WH). The proteins in all the samples analyzed showed an EAAI similar to that of soybean, but higher than that of other oilseeds, such as rapeseed (51). The Protein Efficiency Ratio (PER) showed high values for all the samples analyzed, being similar to those of milk, eggs and soybean (52). The highest PER value was found in GH proteins, and the lowest in WH. Values higher than the standard of 2.0 (53), found for WH, DH and GH samples, indicate that proteins from these sources can be considered of high quality (54).

The predicted biological value (P-BV), which reflects the proportion of absorbed protein that is incorporated into body proteins, recorded values above 90% for all the samples analyzed. However, the P-BV was slightly lower (86.56–88.85%) when the reference standard considered was the AA requirements for growing pigs. However, the values found in this study for P-BV are superior to those reported for peas, chickpeas, beans (55) and white lupin (8).

The Lys/Arg ratio is used to assess the cholesterolemic and atherogenic effects of proteins; a lower ratio suggests a reduced effect on the cardiovascular system (51). In the present study, the Lys/Arg ratio found in all the analyzed samples ranged from 0.35 to 0.37, much lower than that reported for other protein sources frequently consumed by humans, such as milk (2.23), egg (1.05), and soy (0.73) (52).

**Figure 2:**
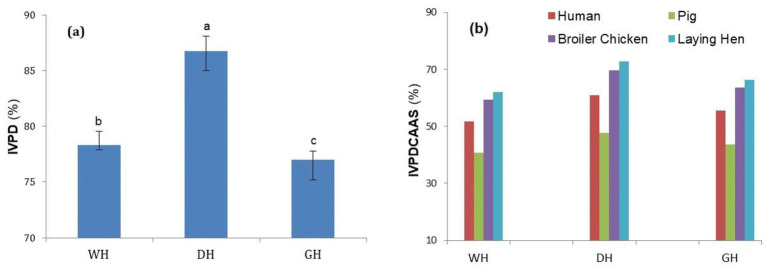
*In vitro* protein digestibility (IVPD) **(a)** and *in vitro* protein digestibility corrected amino acid score (IVPDCAAS) **(b)** of whole (WH), dehulled (DH), and germinated (GH) hemp seeds for different species. Data are plotted as mean ± SD (*n* = 3). Statistical analysis was conducted using one-way ANOVA followed by Duncan’s multiple range test. Different letters above the bars indicate significant differences (*p* < 0.05).

For a correct assessment of protein quality, in addition to individual AA concentrations, protein digestibility should also be considered (38). Proteins from DH samples demonstrated significantly higher *in vitro* digestibility (IVPD) (*p* < 0.01) compared to those from WH and GH samples (Figure 2). The lower IVPD values for WH and GH are probably due to the high crude fiber content that limited protein digestibility (8). Even though GH had a significantly higher (*p* < 0.05) crude fiber content, IVPD did not differ from WH, probably due to the significantly lower (*p* < 0.001) phytic acid and tannin contents of the GH samples (11). Similar results were previously reported by Beltran-Orozco et al. (56), who showed a reduction in IVPD in germinated chia seeds, possibly due to increased fiber and phenolic compounds. These findings contrast with those of a similar study that found a 4.8% increase in IVPD in germinated chia seeds (57). This could have been due to different germination conditions and different origins of the seeds.

The IVPD values found in this study for WH and DH samples (78.34 and 86.73%, respectively) were lower than the digestibility determined *in vivo* (87.01 and 91.88%, respectively) by Nosworthy et al. (10). The IVPDCAAS (*in vitro* protein digestibility corrected amino acid score) combines the concentration of individual amino acids with IVPD, providing a more accurate assessment of protein quality for human and animal nutrition. However, it may be limited by the shortcomings of *in vitro* digestibility tests (11). The IVPDCAAS of the samples investigated in this study ranged from 40.74% for WH protein to 72.77% for DH protein (DH˃GH˃WH). On the other hand, the IVPDAAS values for all the investigated samples were higher when the reference standard was the amino acid requirement of poultry (broilers or laying hens) compared to the values found when the FAO/WHO (24) recommendations or the AA requirements of growing pigs were used as the standard (Figure 2). These findings suggest that hemp proteins are much better suited for poultry feed, followed by the adult human diet, and WH proteins are the least suitable as a source of essential AA for growing pigs, for which the lowest IVPDCAAS values were found (Figure 2). The higher IVPDCAAS value found in GH samples compared to WH is due to the higher amino acid content, even though the IVPD value was not affected by the germination process.

A study by Wang et al. (50) reported IVPDCAAS values ranging from 51 to 64% for WH proteins, which are higher than those found in the present study. For the determination of IVPD, Wang et al. (50) used a different methodology than that used in the present study (different digestive enzymes and a longer digestion time), which would explain the higher IVPDCAAS obtained. The results obtained in this study were partially confirmed by House et al. (38), who reported a PDCAAS score for WH of 51, and 66% for DH, respectively, using the FAO/WHO (27) recommendations as a standard. It is worth noting that protein digestibility was determined *in vivo* in the aforementioned study but *in vitro* in our study.

Hulling was the most effective treatment for improving protein quality parameters (AAS, EAAI, BV, and IVPDCAAS), followed by germination, suggesting that it is more advantageous to use hulled seeds for the development of value-added functional foods.

### 3.4 Fatty acid profiles and their nutritional significance

The predominant FA identified in the investigated samples were linoleic (C18:2n-6, LA), *α*-linolenic (C18:3n-3, ALA), oleic (C18:1c9, OA), palmitic (C16:0, PA), and stearic (C18:0, SA) acids (Table 4).

**Table 4:** Fatty acid (FA) profile in whole (WH), dehulled (DH) and germinated (GH) hemp seeds.

Fatty acids	% of total FA		
	WH	DH	GH
C12:0	0.16 ± 0.02	0.15 ± 0.03	0.13 ± 0.01
C14:0	0.08 ± 0.00	0.06 ± 0.00	0.08 ± 0.01
C16:0 (PA)	6.34 ± 0.71	5.91 ± 0.42	6.63 ± 0.84
C18:0 (SA)	2.26 ± 0.07^b^	2.28 ± 0.08^b^	2.91 ± 0.03^a^
C20:0	0.63 ± 0.03^b^	0.66 ± 0.05^b^	1.02 ± 0.03^a^
Total SFA	9.47 ± 0.45^b^	9.06 ± 0.28^b^	10.77 ± 0.36^a^
C16:1	0.18 ± 0.05^a^	0.17 ± 0.06^a^	0.11 ± 0.02^b^
C18:1 c9 (OA)	11.75 ± 0.87^a^	12.08 ± 1.25^a^	7.17 ± 1.53^b^
Total MUFA	11.93 ± 1.60^a^	12.25 ± 1.82^a^	7.68 ± 1.75^b^
C18:2 n-6 (LA)	54.86 ± 3.21^ab^	53.23 ± 2.47^b^	56.10 ± 2.62^a^
C18:3 n-6 (GLA)	2.31 ± 0.09	2.37 ± 0.13	2.18 ± 0.11
C20:2 n-6	0.27 ± 0.04	0.25 ± 0.02	0.25 ± 0.03
Total n-6 PUFA	57.44 ± 3.72^a^	55.85 ± 2.84^b^	58.53 ± 3.14^a^
C18:3 n-3 (ALA)	19.85 ± 0.35^b^	21.44 ± 0.41^a^	22.31 ± 0.22^a^
C18:4 n-3	0.62 ± 0.03	0.60 ± 0.05	0.56 ± 0.02
C22:5 n-3 (DPA)	0.21 ± 0.02^a^	0.20 ± 0.02^a^	0.15 ± 0.03^b^
C22:6 n-3 (DHA)	0.04 ± 0.01	0.05 ± 0.01	0.04 ± 0.01
Total n-3 PUFA	20.72 ± 0.41^b^	22.29 ± 0.57^a^	23.06 ± 0.39^a^
Total PUFA	78.16 ± 0.82^b^	78.14 ± 1.06^b^	81.59 ± 1.12^a^
Other FA	0.43 ± 0.04	0.55 ± 0.03	0.36 ± 0.06

SFA, saturated FA; MUFA, monounsaturated FA; PUFA, polyunsaturated FA; PA, palmitic acid; SA, stearic acid; OA, oleic acid; LA, linoleic acid; GLA, gamma linolenic acid; ALA, α-linolenic acid; DPA, docosapentaenoic acid; DHA, docosahexaenoic acid. Data are presented as mean ± standard deviation (SD) (*n* = 3). Values within a row not marked with the same superscript letter indicate significant differences according to Duncan’s multiple range test (*p* < 0.05).

The main FA found in hulled seeds were similar to those found in whole hemp seeds, but in different proportions, due to the removal of the hull, which does not contain significant amounts of FA (58). Compared to whole seeds, hulled seeds had a higher content of ALA and of total n-6 PUFA. On the other hand, germinated samples had higher contents of LA, ALA and SA, but lower OA, compared to whole seeds.

Hemp seeds are recognized as a rich source of omega-3, considered beneficial for human health (35). The results obtained in this study indicated a significant increase in omega-3 content after hulling or germination, while the omega-6 content increased significantly only in germinated seeds. The recommended daily intake of ALA and LA is 0.5 and 4% of total daily energy requirements, respectively (58), corresponding to 1.1 g of ALA and 8.9 g of LA. The results of the present study indicate that the daily requirement for ALA can be met by consuming only 11 g of DH or 18 g of WH or GH, while meeting the daily requirement for LA requires a larger amount of seeds: 35 g for DH, 52 g for WH, and 60 g for GH. The smaller quantity of DH needed to meet the recommended daily intake of ALA and LA highlights the higher lipid density of dehulled seeds, resulting from the removal of the hull, which does not contain significant amounts of fatty acids.

In this context, hulled or germinated hemp seeds, through their increased content of omega-3, can significantly contribute to improving health, given their potential to reduce the risk of cardiovascular disease, reduce inflammation, and prevent and control neurodegenerative diseases (59). In addition, the use of these sources rich in omega-3 FA in animal feed provides products (eggs, meat, and milk) enriched with omega-3, which can be considered “functional foods” with benefits for human health (3, 32, 33).

Contrary to the results obtained in the present study, Ghafoor et al. (60) reported a reduction in omega-3 FA after chia seed germination, while other studies (40) found no change in the FA profile in germinated chia and flax seeds. On the other hand, sprouting broccoli seeds showed increased omega-3 FA content, similar to our findings (43). These differences could have occurred due to the different chemical composition and fatty acid profile, but also the germination time (40).

Sources of *γ*-linolenic acid (C18:3n-6, GLA) are limited, and hemp seeds are an important source of GLA for the human diet. In the present study, the GLA concentration was not affected by the hulling or germination of the seeds. GLA is important for health because it contributes to the formation of anti-inflammatory eicosanoids, which have anticancer, vasodilating and cholesterol-lowering effects (53).

The samples investigated in this study presented fat quality indices (AI, TI, h/H, n-6/n-3 FA) similar to those of hemp seeds, flax, chia, quinoa and pumpkin (59). The lower values for AI and TI and higher values for h/H and HPI found in the DH samples (Table 5) suggest an improvement in the quality of fats in hemp seeds after hulling and their great potential to prevent cardiovascular diseases (59).

**Table 5:** Fatty acid ratios and quality indices of lipids in whole (WH), dehulled (DH) and germinated (GH) hemp seeds.

Parameters	WH	DH	GH
SFA/UFA	0.11 ± 0.02	0.10 ± 0.02	0.12 ± 0.03
PUFA/SFA	8.25 ± 0.25^a^	8.62 ± 0.37^a^	7.57 ± 0.15^b^
n-6/n-3 PUFA	2.77 ± 0.10	2.51 ± 0.08	2.54 ± 0.17
Atherogenic index (AI)	0.076 ± 0.01^a^	0.069 ± 0.01^b^	0.079 ± 0.02^a^
Thrombogenic index (TI)	0.089 ± 0.01^ab^	0.081 ± 0.03^b^	0.094 ± 0.05^a^
Peroxidizability index (PI)^*^	103.81 ± 0.70	105.41 ± 0.85	104.31 ± 0.57
hipo/Hipercholesterolemic FA (h/H)	13.66 ± 0.45^ab^	14.74 ± 0.32^a^	12.97 ± 0.28^b^
Oxidative stability (OS)	4,553 ± 29	4,637 ± 43	4,343 ± 21
Health-promoting Index (HPI)	13.21 ± 0.66^ab^	14.35 ± 0.21^a^	12.61 ± 0.54^b^
Nutritive Value Indices (NVI)	2.21 ± 0.13^a^	2.43 ± 0.09^a^	1.52 ± 0.07^b^

* PI = monoenoic acid x 0.025 + dienoic acid x 1 + trienoic acid x 2 + tetranoic acid x 4 + pentanoic acid x 6 + hexanoic acid x 8. Data are presented as mean ± standard deviation (SD) (*n* = 3). Values within a row not marked with the same superscript letter indicate significant differences according to Duncan’s multiple range test (*p* < 0.05).

### 3.5 Tocopherol contents

Hulling resulted in a significant increase in *α*-, *γ*- and *δ*-tocopherol content, and germination increased α-tocopherol levels more than fourfold compared to whole seeds, while the γ- and δ-tocopherol content decreased (*p* < 0.05) (Table 6). In all three samples investigated (WH, DH, and GH), γ-tocopherol was the predominant isomer, in agreement with previous studies showing that γ-tocopherol represents more than 90% of the total tocopherol content in hemp seeds and hemp oil (1, 42, 58).

**Table 6:** Tocopherol contents of whole (WH), dehulled (DH) and germinated (GH) hemp seeds (mg/100 g DM of seed).

Hemp seeds	*α*-Tocopherol	γ-Tocopherol	δ-Tocopherol	Total tocopherols
WH	1.89 ± 0.16^c^	22.21 ± 0.43^b^	0.93 ± 0.08^b^	25.03 ± 0.58^b^
DH	2.52 ± 0.11^b^	30.17 ± 0.65^a^	1.86 ± 0.20^a^	34.65 ± 0.84^a^
GH	7.83 ± 0.58^a^	16.71 ± 0.26^c^	1.53 ± 0.12^a^	26.07 ± 0.43^b^

Data are presented as mean ± standard deviation (SD) (*n* = 3). Values within a row not marked with the same superscript letter indicate significant differences according to Duncan’s multiple range test (*p* < 0.05).

The total tocopherol content of 34.6 mg/100 g found in this study for DH is similar to that previously reported by Alonso-Esteban et al. (58) of 36.8 mg/100 g, but it is strikingly higher than the average reported by Hwang et al. (1) of 27.8 mg/100 g. These variations can be attributed to genotype and environmental factors that can influence the tocopherol content of hemp seeds (7). Contrary to the results of our study, Kundgol et al. (61) observed a reduction in α-tocopherol content upon hulling millet seeds.

In agreement with the present study, research by Tarasevičienė et al. (62) confirmed that, after seed germination for 5 days, the content of α-tocopherol increased by 5.2-fold (in wheat) to 6.9-fold (in alfalfa), and that of γ- and δ-tocopherol decreased. However, Fernandez-Orozco et al. (63) found that, after germination, γ-tocopherol increased in soybeans, and decreased in germinated lupin and lentil grains; Tarasevičienė et al. (62) concluded that the mechanisms involved in the modification of tocopherol content during seed germination were still unclear.

### 3.6 Total polyphenols and total flavonoid content

**Figure 3:**
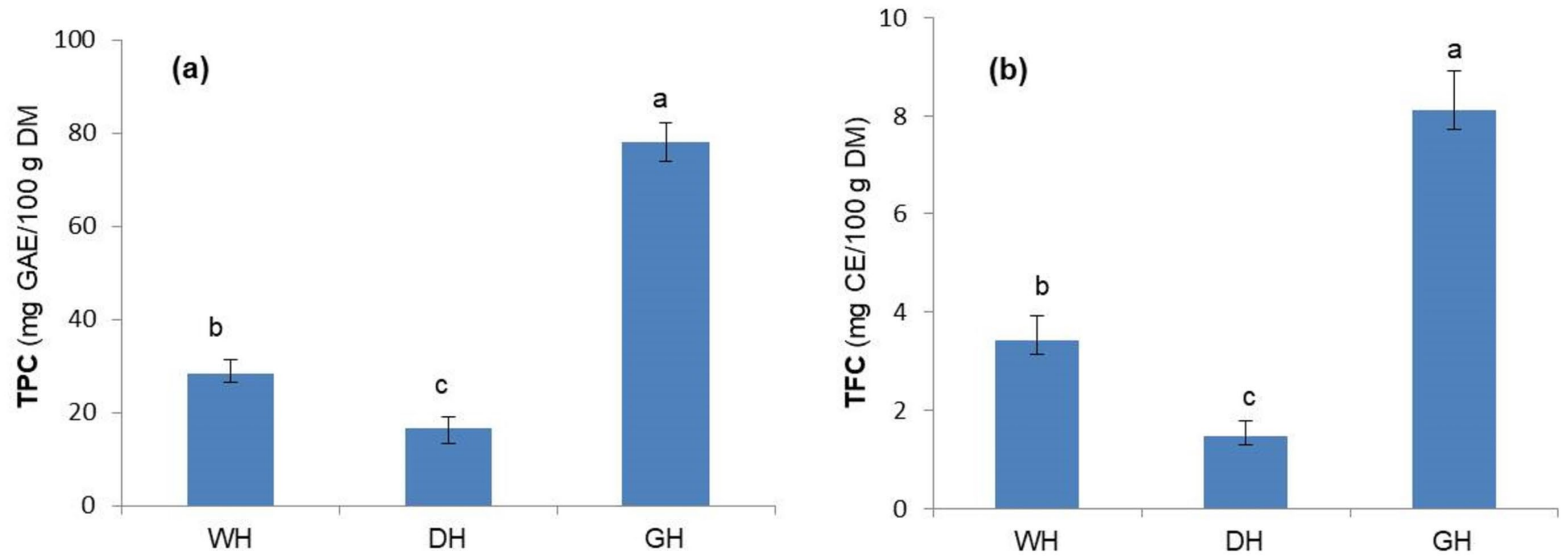
Total phenolic content (TPC) **(a)** and total flavonoid content (TFC) **(b)** of whole (WH), dehulled (DH), and germinated (GH) hemp seeds. GAE, Gallic acid equivalents; CE, catechin equivalents; TE, Trolox equivalents. Data are plotted as mean ± SD (*n* = 3). Statistical analysis was conducted using one-way ANOVA followed by Duncan’s multiple range test. Different letters above the bars indicate significant differences (*p* < 0.05).

Dehulling hemp seeds significantly reduced the total phenolic content (TPC), from 28.32 mg GAE/100 g DM to 16.74 mg GAE/100 g DM (Figure 3), confirming the results of previous studies (12) showing that most polyphenols are concentrated in the seed coats, and that dehulling reduces polyphenols in dehulled seeds. A similar reduction was also observed for the total flavonoid content (TFC), which decreased by approximately half after dehulling hemp seeds (3.41 vs. 1.47 mg CE/100 g DM) (Figure 3). This decrease in TFC is not surprising, as flavonoids are a subclass of polyphenols and, therefore, decreased with a similar trend to TPC (12). Similarly, the hulling of bean seeds or horsegram seeds reduced their polyphenol content by up to 52% (14).

The TPC found in the present study for WH (28.32 mg GAE/100 g) was partially in agreement with Hwang et al. (1), who showed that, depending on the hemp genotype and cultivation region, the TPC values ranged from 28.16 mg GAE/100 g to 46.33 mg GAE/100 g. In addition, Nounah et al. (64) demonstrated that environmental conditions, such as precipitation and temperature, can significantly influence the polyphenol content of plant seeds, suggesting that relatively dry and warm conditions may induce stress responses that favor the accumulation of polyphenols in seeds.

Germination increased TPC by 2.76-fold and TFC by 2.38-fold compared to WH. Although similar increasing trends have been reported for other species, such as chia (40), lentil (41), sunflower, broccoli (65), radish, and alfalfa (62), the magnitude of these changes appears to be highly variable in the literature. For example, the increases observed in the present study are lower than the 6.4- and 11.5-fold increases reported for germinated chia seeds (16). In contrast, other studies have even reported decreases in phenolic compound content in lentil, alfalfa (39), and horse gram sprouts (14). These discrepancies suggest that relative increases should be interpreted with caution, as they largely depend on the initial phenolic compound content of dormant seeds, which is significantly influenced by genotype and environmental factors such as temperature and precipitation levels (66). In addition, methodological differences in extraction or losses during seed soaking likely contribute to the inconsistent trends observed across studies. The increase in polyphenol content in germinated seeds may be related to the activation of endogenous enzymes and the complex biochemical metabolism of the seeds during this process (65). Concurrently, *de novo* biosynthesis of phenolic compounds occurs in response to the stress initiated by the germination process (16).

The increases in the contents of phenolic compounds and flavonoids in GH samples may be associated with an improvement in the functional properties of germinated hemp seeds, as these bioactive compounds have been associated with a reduced risk of cardiovascular disease, hepatoprotective effects, and protection against plasma oxidative stress (15).

### 3.7 Antioxidant activity

It is important that at least two methods based on different reaction mechanisms be used to evaluate antioxidant activity (67). In our study, the antioxidant activity was evaluated using the ability to capture the radical cation of the diammonium salt ABTS• + (2,2′-azino-bis(3-ethylbenzothiazoline-6-sulfonic acid)) and the “stable” free radical DPPH• (2,2 diphenyl-1-picrylhydrazyl).

**Figure 4:**
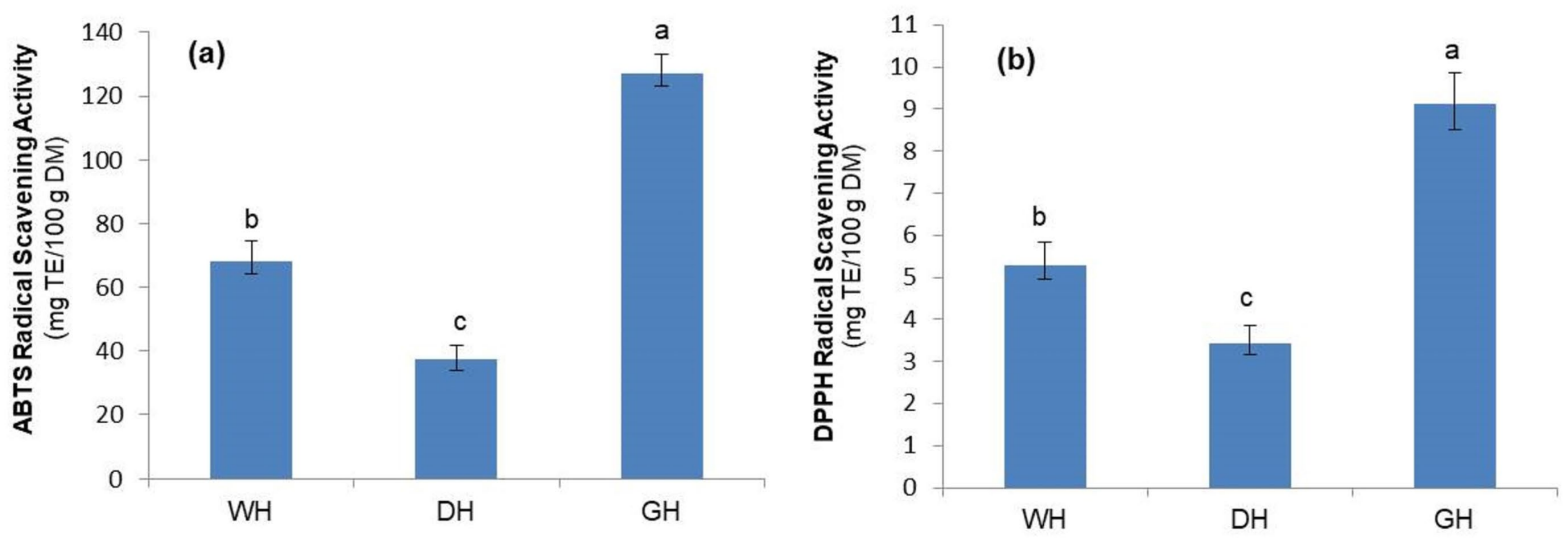
Antioxidant activity of whole (WH), dehulled (DH), and germinated (GH) hemp seeds, measured by ABTS assays **(a)** and DPPH assays **(b)**; TE: Trolox equivalents. Data are plotted as mean ± SD (*n* = 3). Statistical analysis was conducted using one-way ANOVA followed by Duncan’s multiple range test. Different letters above the bars indicate significant differences (*p* < 0.05).

The results of the two tests used (ABTS and DPPH) (Figure 4) show that the GH samples had a higher antioxidant activity (*p* < 0.05) compared to the WH, and the lowest antioxidant activity was in the DH samples (*p* < 0.05). The increased antioxidant activity of the GH samples found in this study can be attributed to the increased concentrations of antioxidant compounds such as *α*-tocopherol, phenolic compounds and flavonoids, in addition to the enzymatic hydrolysis of conjugated phenolic compounds into free phenolic compounds, which are more bioavailable and biologically active (66). This finding was confirmed by Frassinetti et al. (17), who showed a significant linear correlation between the polyphenol content and antioxidant activity of hemp sprouts, assessed *in vitro* by the DPPH radical-scavenging activity (*r* = 0.88). Positive effects of germination on antioxidant activity have also been demonstrated in other plant species, such as chia seeds (40), sunflower, alfalfa (62), radish and broccoli (65).

In line with this study, a previous study found that WH showed better antioxidant potential compared to DH (12). This is related to the higher content of phenolic compounds in WH samples, which provides them with higher antioxidant activity than the dehulled variants. Similarly, dehulling horsegram seeds significantly reduced the concentrations of phytochemicals responsible for antioxidant activity (14).

There were strong correlations between the TPC and TFC and antioxidant activity as assessed by the ABTS assay (*r* = 0.969 and 0.978, respectively) and a weaker but statistically significant correlation (*p* <0.05) between the TPC and TFC and antioxidant activity as assessed by the DPPH assay (*r* = 0.827 and 0.828, respectively). The smallest, but significant (*p* < 0.05), correlations were observed between *α*-tocopherol content and antioxidant activity as assessed by the ABTS and DPPH assays (*r* = 0.812 and 0.791, respectively) (Table 7). These results suggest that phenolic compounds are better scavengers of ABTS radicals than of DPPH radicals, while α-tocopherol has a lower ABTS radical-scavenging potential than phenolic compounds. These findings are partially confirmed by Beltran-Orozco et al. (56), who, in agreement with our study, found strong correlations between the TPC and TFC and antioxidant activity of chia seeds as assessed using the ABTS assay, but, on the other hand, reported higher correlations between TPC and TFC and antioxidant activity as assessed by the DPPH assay. These differences may be related to the fact that radical-scavenging activity depends on the species, germination time, and qualitative and quantitative contents of antioxidant compounds (62).

**Table 7:** Coefficients of correlations between antioxidant compounds and the ABTS and DPPH radical-scavenging activities.

Antioxidant compounds	Antioxidant capacity assays	
	ABTS	DPPH
Total phenolic content	0.969^*^	0.827^*^
Total flavonoid content	0.978^*^	0.828^*^
α-tocopherol	0.812^*^	0.791^*^

* Correlation significant at the *p* < 0.05 level.

Strong correlations (*r* > 0.9) between polyphenol/flavonoid content and antioxidant capacity indicate that TPC and TFC can be used as rapid and cost-effective predictive indicators for monitoring the antioxidant potential of hemp seeds, without the need to perform more complex and expensive radical scavenging assays (ABTS/DPPH). The validation of polyphenols as the main antioxidant agents through these strong correlations supports the incorporation of hemp seeds into fat-rich food matrices to prevent lipid peroxidation and extend shelf life, thereby meeting consumer demand for “clean label” products. Differences in correlation between ABTS and DPPH suggest that producers must select antioxidant markers and evaluation methods tailored to the specific hydrophilic or lipophilic nature of hemp-enriched foods, in order to ensure oxidative stability and maximize the nutritional benefits of the final product.

It is known that unsaturated FA, and especially omega-3, are highly susceptible to oxidation (31, 32). In the present study, seed hulling significantly increased the omega-3 content, while the contents of phenolic compounds and flavonoids decreased, suggesting an increased risk of lipid oxidation in DH samples. A similar effect of increasing the omega-3 content was observed in germinated seeds, but germination also increased the contents of antioxidants (TPC, TFC and *α*-tocopherol) and, implicitly, the antioxidant activity. Consequently, germination improved the functional properties of hemp seeds as antioxidants, which can be used to obtain functional foods, with implications for alleviating oxidative stress, preventing free radical damage and improving human health (66). For animal nutrition, germinated seeds could be recommended for laying hens, due to their high antioxidant activity, which can ensure greater oxidative stability of the yolk during egg storage (3), while dehulled seeds could be recommended for feeding broiler chickens intended for the production of fresh meat enriched with fatty acids of nutritional interest. *In vivo* tests will be necessary to evaluate the efficacy of dehulled or germinated hemp seeds in promoting animal health, productive performance and product quality (eggs and meat).

## 4 Conclusions and perspectives

This study compared the nutritional and antinutritional components, amino acid and fatty acid profiles, bioactive components and antioxidant activity of whole, hulled and germinated hemp seeds.

Dehulled seeds demonstrated the highest macronutrient density, characterized by a significantly higher content of protein and oil. The removal of the hull, where most insoluble fiber and compounds with antinutritional effects are concentrated, ensured the highest *in vitro* protein digestibility, reaching levels comparable to animal proteins. At the same time, dehulled seeds exhibited a more nutritionally favorable profile of essential amino acids and fatty acids compared to whole or germinated seeds. In all the samples investigated, the first limiting amino acid was lysine, for both humans and animals, which highlights the need to include lysine content in future hemp-breeding programs.

Although sprouting reduced total lipids, it increased omega-3 FA, which play essential roles in cardiovascular and brain health. In addition, hemp sprouts demonstrated greater antioxidant activity, due to increased concentrations of antioxidant compounds such as *α*-tocopherol, polyphenols and flavonoids.

In the context of the growing interest in plant-based protein diets, our results suggest that hulled or germinated hemp seeds have the potential to serve as sustainable sources of protein and bioactive compounds (omega-3 and antioxidants). However, confirmation of their capacity to promote health requires validation through clinical research. Future studies should focus on optimizing germination conditions and, crucially, on evaluating nutrient bioavailability using *in vivo* models. Additionally, the potential use of processed hemp in poultry diets should be validated through long-term *in vivo* trials in order to confirm the efficiency of omega-3 and antioxidant transfer into eggs and meat, as well as the oxidative stability of these products during storage.

## Data Availability

The original contributions presented in the study are included in the article/supplementary material, further inquiries can be directed to the corresponding author.
